# *catena*-Poly[[[1,2-bis­(benzimidazol-2-yl)ethane]cadmium(II)]-μ-sebacato]

**DOI:** 10.1107/S1600536810005714

**Published:** 2010-03-13

**Authors:** Yan-Ling Zhou, Hong Liang, Ming-Hua Zeng

**Affiliations:** a School of Chemistry and Chemical Engineering, Central South University, Changsha 410083, People’s Republic of China; b School of Chemistry and Chemical Engineering, Guangxi Normal University, Guilin, 541004, People’s Republic of China

## Abstract

In the title compound, [Cd(C_10_H_16_O_4_)(C_16_H_14_N_4_)]_*n*_, the Cd^II^ ion is six-coordinated in a distorted octa­hedral geometry by four carboxyl­ate O atoms from two sebacate ligands and two N atoms from the chelating 1,4-bis­(2-benzimidazol­yl)ethanebutane ligand. Neighboring Cd^II^ ions are bridged by the sebacate ligands, forming a zigzag polymeric chain structure. The chains are further extended into a three-dimensional supra­molecular structure through inter­molecular N—H⋯O hydrogen bonds.

## Related literature

For the synthesis of the ligand, see: van Albada *et al.* (1995 ▶) and literature cited therein. For *M*–dicarboxyl­ate complexes with aromatic *N*-donor chelating ligands, see: Wei *et al.* (2010 ▶) [*M* = lead(II) adduct]; Meng *et al.* (2008 ▶) [*M* = zinc(II) adduct]; Wang *et al.* (2006 ▶) [*M* = cadmium(II) and zinc(II) adducts].
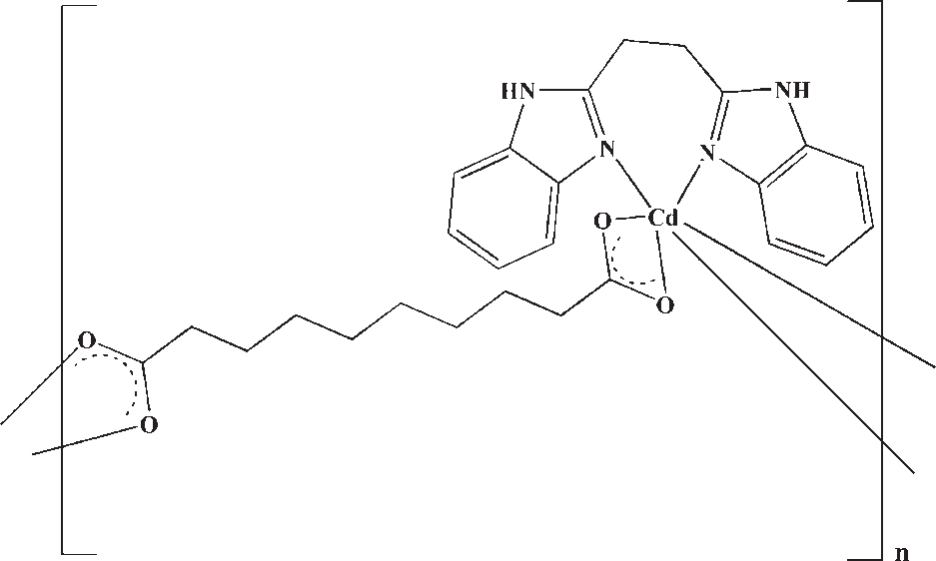


## Experimental

### 

#### Crystal data

[Cd(C_10_H_16_O_4_)(C_16_H_14_N_4_)]
*M* *_r_* = 574.95
Monoclinic, 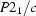

*a* = 8.7554 (14) Å
*b* = 15.674 (3) Å
*c* = 18.455 (3) Å
β = 98.851 (3)°
*V* = 2502.4 (7) Å^3^
*Z* = 4
Mo *K*α radiation
μ = 0.91 mm^−1^
*T* = 110 K
0.48 × 0.34 × 0.27 mm

#### Data collection

Bruker SMART APEX CCD area-detector diffractometer
Absorption correction: multi-scan (*SADABS*; Sheldrick, 1996 ▶) *T*_min_ = 0.669, *T*_max_ = 0.791
10665 measured reflections
4390 independent reflections
3533 reflections with *I* > 2σ(*I*)
*R*_int_ = 0.026

#### Refinement

*R*[*F* ^2^ > 2σ(*F* ^2^)] = 0.032
*wR*(*F* ^2^) = 0.096
*S* = 1.09
4390 reflections
316 parameters
H-atom parameters constrained
Δρ_max_ = 0.82 e Å^−3^
Δρ_min_ = −0.44 e Å^−3^

### 

Data collection: *SMART* (Bruker, 2001 ▶); cell refinement: *SAINT* (Bruker, 2001 ▶); data reduction: *SAINT*; program(s) used to solve structure: *SHELXS97* (Sheldrick, 2008 ▶); program(s) used to refine structure: *SHELXL97* (Sheldrick, 2008 ▶); molecular graphics: *X-SEED* (Barbour, 2001 ▶); software used to prepare material for publication: *publCIF* (Westrip, 2010 ▶) and *PLATON* (Spek, 2009 ▶).

## Supplementary Material

Crystal structure: contains datablocks global, I. DOI: 10.1107/S1600536810005714/si2241sup1.cif

Structure factors: contains datablocks I. DOI: 10.1107/S1600536810005714/si2241Isup2.hkl

Additional supplementary materials:  crystallographic information; 3D view; checkCIF report

## Figures and Tables

**Table 1:** Selected bond lengths (Å)

Cd1—N1	2.246 (3)
Cd1—N3	2.287 (3)
Cd1—O2	2.340 (3)
Cd1—O1	2.348 (3)
Cd1—O3	2.377 (3)
Cd1—O4	2.382 (3)

**Table 2:** Hydrogen-bond geometry (Å, °)

*D*—H⋯*A*	*D*—H	H⋯*A*	*D*⋯*A*	*D*—H⋯*A*
N2—H2*A*⋯O2^i^	0.88	1.85	2.722 (4)	173
N4—H4*A*⋯O3^ii^	0.88	1.87	2.686 (4)	154

Symmetry codes: (i) 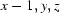; (ii) 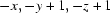.

## supplementary crystallographic information

### Comment

In recent years, studies on metal-dicarboxylate complexes with aromatic N-donor chelating ligands have attracted special attention because of their interesting structural and chemical properties (Wei *et al.*, 2010; Meng *et al.*, 2008; Wang *et al.*, 2006). Herein, the title new cadmium-dicarboxylate complex, Fig. 1, is reported.

Selected bond distances are listed in Table 1. Each Cd(II) center is six-coordinated by two N atoms of the chelating 1,4-Bis(2-benzimidazolyl)ethanebutane ligand and four O atoms from two sebacate ligands. The neighboring Cd(II) ions are bridged by sebacate ligands to form a zigzag polymeric chain structure (Fig. 2). In the crystalstructure, the adjacent chains are linked via N—H···O hydrogen bonds (Table 2) resulting in the formation of a three-dimensional supramolecular structure.

### Experimental

1,4-Bis(2-benzimidazolyl)ethanebutane was synthesized by using aliterature method (van Albada *et al.*, 1995). A solution of Cd(NO_3_)_2_.6H_2_O (0.17 g, 0.5 mmol), 1,4-Bis(2-benzimidazolyl)ethanebutane (0.13 g, 0.5 mmol), sebacic acid (0.10 g, 0.5 mmol), NaOH (0.02 g, 1 mmol) in H_2_O (10 ml) and CH_3_OH (5 ml) was stirred under ambient conditions, then sealed in a Teflon-lined steel vessel, heated at 443 K for 3 d, and cooled to room temperature. The resulting product was recovered by filtration, washed with distilled water and dried in air (35% yield).

### Refinement

The C-bound H atoms were placed in calculated positions (C—H = 0.95–0.98 Å) and included in the refinement in the riding-model approximation, with *U*_iso_(H) = 1.2*U*_eq_(C). The amino H atoms were located in a difference Fourier map and refined isotropically with distance restraints of N—H = 0.88 (1) Å.

### Crystal data

**Table:**

[Cd(C_10_H_16_O_4_)(C_16_H_14_N_4_)]	*F*(000) = 1176
*M**_r_* = 574.95	*D*_x_ = 1.526 Mg m^−^^3^
Monoclinic, *P*2_1_/*c*	Mo *K*α radiation, λ = 0.71073 Å
Hall symbol: -P 2ybc	Cell parameters from 5481 reflections
*a* = 8.7554 (14) Å	θ = 2.4–27.0°
*b* = 15.674 (3) Å	µ = 0.91 mm^−^^1^
*c* = 18.455 (3) Å	*T* = 110 K
β = 98.851 (3)°	Block, yellow
*V* = 2502.4 (7) Å^3^	0.48 × 0.34 × 0.27 mm
*Z* = 4	

### Data collection

**Table:**

Bruker SMART APEX CCD area-detector diffractometer	4390 independent reflections
Radiation source: fine-focus sealed tube	3533 reflections with *I* > 2σ(*I*)
graphite	*R*_int_ = 0.026
phi and ω scans	θ_max_ = 25.0°, θ_min_ = 1.7°
Absorption correction: multi-scan (*SADABS*; Sheldrick, 1996)	*h* = −10→6
*T*_min_ = 0.669, *T*_max_ = 0.791	*k* = −18→17
10665 measured reflections	*l* = −21→21

### Refinement

**Table:**

Refinement on *F*^2^	Primary atom site location: structure-invariant direct methods
Least-squares matrix: full	Secondary atom site location: difference Fourier map
*R*[*F*^2^ > 2σ(*F*^2^)] = 0.032	Hydrogen site location: inferred from neighbouring sites
*wR*(*F*^2^) = 0.096	H-atom parameters constrained
*S* = 1.09	*w* = 1/[s^2^(*F*_o_^2^) + (0.048*P*)^2^ + 3.287*P*] where *P* = (*F*_o_^2^ + 2*F*_c_^2^)/3
4390 reflections	(Δ/σ)_max_ < 0.001
316 parameters	Δρ_max_ = 0.82 e Å^−^^3^
0 restraints	Δρ_min_ = −0.44 e Å^−^^3^

### Special details

**Table:**

Refinement. Refinement of *F*^2^ against ALL reflections. The weighted *R*-factor wR and goodness of fit *S* are based on *F*^2^, conventional *R*-factors *R* are based on *F*, with *F* set to zero for negative *F*^2^. The threshold expression of *F*^2^ > σ(*F*^2^) is used only for calculating *R*-factors(gt) etc. and is not relevant to the choice of reflections for refinement. *R*-factors based on *F*^2^ are statistically about twice as large as those based on *F*, and *R*- factors based on ALL data will be even larger.

### Fractional atomic coordinates and isotropic or equivalent isotropic displacement parameters (Å^2^)

**Table:**

	*x*	*y*	*z*	*U*_iso_*/*U*_eq_	
Cd1	0.09748 (3)	0.490930 (16)	0.300044 (14)	0.01805 (11)	
N1	−0.1515 (4)	0.52080 (19)	0.25864 (16)	0.0194 (7)	
N2	−0.4062 (4)	0.50949 (19)	0.25203 (17)	0.0203 (7)	
H2A	−0.4966	0.4885	0.2581	0.024*	
N3	0.0647 (4)	0.41909 (19)	0.40440 (16)	0.0181 (7)	
N4	−0.0190 (3)	0.38418 (19)	0.50792 (15)	0.0190 (7)	
H4A	−0.0836	0.3724	0.5387	0.023*	
O1	0.1064 (3)	0.38258 (17)	0.21291 (15)	0.0269 (6)	
O2	0.3259 (3)	0.43106 (16)	0.27181 (14)	0.0210 (6)	
O3	0.1740 (3)	0.60960 (16)	0.37739 (13)	0.0217 (6)	
O4	0.1927 (3)	0.62282 (16)	0.26098 (14)	0.0233 (6)	
C1	−0.2210 (4)	0.5909 (2)	0.2205 (2)	0.0214 (8)	
C2	−0.1571 (5)	0.6594 (3)	0.1866 (2)	0.0278 (9)	
H2B	−0.0485	0.6645	0.1883	0.033*	
C3	−0.2570 (5)	0.7191 (3)	0.1510 (2)	0.0299 (10)	
H3A	−0.2164	0.7663	0.1278	0.036*	
C4	−0.4171 (5)	0.7116 (3)	0.1481 (2)	0.0302 (10)	
H4B	−0.4822	0.7544	0.1234	0.036*	
C5	−0.4836 (5)	0.6447 (3)	0.1799 (2)	0.0272 (9)	
H5A	−0.5924	0.6396	0.1773	0.033*	
C6	−0.3813 (4)	0.5844 (2)	0.2163 (2)	0.0213 (8)	
C7	−0.2672 (4)	0.4744 (2)	0.27599 (19)	0.0197 (8)	
C8	−0.2521 (5)	0.3929 (2)	0.3185 (2)	0.0219 (8)	
H8A	−0.1653	0.3598	0.3040	0.026*	
H8B	−0.3476	0.3591	0.3045	0.026*	
C9	−0.2244 (4)	0.4032 (2)	0.4013 (2)	0.0206 (8)	
H9A	−0.2728	0.4573	0.4137	0.025*	
H9B	−0.2777	0.3561	0.4230	0.025*	
C10	−0.0578 (4)	0.4038 (2)	0.4366 (2)	0.0191 (8)	
C11	0.1930 (4)	0.4077 (2)	0.46061 (19)	0.0176 (8)	
C12	0.3508 (4)	0.4136 (2)	0.4588 (2)	0.0226 (9)	
H12A	0.3884	0.4293	0.4150	0.027*	
C13	0.4501 (5)	0.3963 (2)	0.5213 (2)	0.0224 (8)	
H13A	0.5583	0.3992	0.5209	0.027*	
C14	0.3941 (5)	0.3740 (2)	0.5875 (2)	0.0236 (9)	
H14A	0.4658	0.3629	0.6306	0.028*	
C15	0.2386 (5)	0.3684 (2)	0.5901 (2)	0.0220 (8)	
H15A	0.2001	0.3537	0.6338	0.026*	
C16	0.1396 (4)	0.3857 (2)	0.5243 (2)	0.0189 (8)	
C17	0.2500 (5)	0.3857 (2)	0.2217 (2)	0.0212 (8)	
C18	0.3369 (5)	0.3376 (2)	0.1691 (2)	0.0242 (9)	
H18A	0.4488	0.3499	0.1815	0.029*	
H18B	0.3220	0.2755	0.1749	0.029*	
C19	0.2816 (5)	0.3627 (2)	0.0897 (2)	0.0271 (9)	
H19A	0.3410	0.3303	0.0574	0.033*	
H19B	0.1712	0.3472	0.0764	0.033*	
C20	0.3008 (5)	0.4576 (3)	0.0773 (2)	0.0288 (9)	
H20A	0.4098	0.4733	0.0949	0.035*	
H20B	0.2356	0.4892	0.1075	0.035*	
C21	0.2588 (5)	0.4865 (3)	−0.0021 (2)	0.0285 (9)	
H21A	0.1521	0.4681	−0.0212	0.034*	
H21B	0.3293	0.4589	−0.0322	0.034*	
C22	0.2704 (5)	0.5833 (3)	−0.0092 (2)	0.0305 (10)	
H22A	0.3746	0.6015	0.0142	0.037*	
H22B	0.1944	0.6100	0.0183	0.037*	
C23	0.2422 (5)	0.6172 (2)	−0.0878 (2)	0.0245 (9)	
H23A	0.3248	0.5961	−0.1143	0.029*	
H23B	0.1423	0.5949	−0.1132	0.029*	
C24	0.2393 (5)	0.7145 (2)	−0.0905 (2)	0.0281 (9)	
H24A	0.1541	0.7353	−0.0656	0.042*	
H24B	0.3375	0.7366	−0.0631	0.042*	
C25	0.2180 (5)	0.7502 (2)	0.3325 (2)	0.0225 (8)	
H25A	0.1273	0.7789	0.3040	0.034*	
H25B	0.3096	0.7666	0.3101	0.034*	
C26	0.1956 (4)	0.6554 (2)	0.3231 (2)	0.0210 (8)	

### Atomic displacement parameters (Å^2^)

**Table:**

	*U*^11^	*U*^22^	*U*^33^	*U*^12^	*U*^13^	*U*^23^
Cd1	0.01791 (16)	0.02168 (16)	0.01482 (16)	−0.00217 (11)	0.00329 (10)	0.00041 (11)
N1	0.0207 (17)	0.0248 (17)	0.0121 (16)	−0.0036 (13)	0.0001 (13)	0.0008 (12)
N2	0.0156 (16)	0.0292 (18)	0.0161 (16)	−0.0049 (14)	0.0023 (13)	−0.0007 (13)
N3	0.0228 (17)	0.0168 (15)	0.0149 (16)	−0.0013 (13)	0.0036 (13)	0.0002 (12)
N4	0.0260 (19)	0.0199 (16)	0.0119 (16)	0.0022 (13)	0.0056 (14)	0.0031 (12)
O1	0.0209 (16)	0.0298 (15)	0.0292 (16)	−0.0017 (12)	0.0016 (12)	−0.0059 (12)
O2	0.0210 (14)	0.0223 (14)	0.0199 (14)	−0.0034 (11)	0.0036 (11)	−0.0037 (11)
O3	0.0284 (15)	0.0215 (14)	0.0164 (14)	−0.0031 (11)	0.0073 (11)	0.0033 (11)
O4	0.0301 (16)	0.0256 (14)	0.0146 (14)	−0.0033 (12)	0.0045 (12)	−0.0017 (11)
C1	0.026 (2)	0.022 (2)	0.0157 (19)	−0.0027 (16)	−0.0013 (16)	−0.0016 (15)
C2	0.025 (2)	0.031 (2)	0.027 (2)	−0.0058 (18)	0.0021 (17)	0.0037 (18)
C3	0.039 (3)	0.026 (2)	0.024 (2)	−0.0026 (19)	0.0026 (19)	0.0080 (17)
C4	0.034 (3)	0.029 (2)	0.027 (2)	0.0033 (19)	−0.0005 (19)	0.0054 (18)
C5	0.025 (2)	0.031 (2)	0.025 (2)	0.0014 (18)	0.0011 (17)	−0.0028 (17)
C6	0.025 (2)	0.022 (2)	0.017 (2)	0.0003 (16)	0.0022 (16)	−0.0020 (15)
C7	0.023 (2)	0.023 (2)	0.0126 (19)	−0.0032 (16)	0.0034 (16)	−0.0052 (15)
C8	0.024 (2)	0.022 (2)	0.020 (2)	−0.0056 (16)	0.0026 (16)	−0.0013 (15)
C9	0.023 (2)	0.022 (2)	0.017 (2)	−0.0033 (16)	0.0059 (16)	0.0019 (15)
C10	0.023 (2)	0.0124 (18)	0.022 (2)	−0.0009 (15)	0.0044 (16)	−0.0023 (15)
C11	0.024 (2)	0.0118 (17)	0.0165 (19)	−0.0007 (15)	0.0018 (16)	−0.0010 (14)
C12	0.027 (2)	0.0191 (19)	0.022 (2)	−0.0021 (16)	0.0049 (17)	0.0005 (15)
C13	0.022 (2)	0.022 (2)	0.022 (2)	0.0002 (16)	0.0012 (17)	−0.0042 (16)
C14	0.031 (2)	0.020 (2)	0.017 (2)	0.0055 (17)	−0.0016 (17)	−0.0012 (15)
C15	0.030 (2)	0.020 (2)	0.016 (2)	0.0039 (16)	0.0040 (17)	0.0028 (15)
C16	0.026 (2)	0.0159 (18)	0.0142 (19)	0.0006 (16)	0.0021 (16)	−0.0027 (14)
C17	0.026 (2)	0.0192 (19)	0.018 (2)	−0.0037 (16)	0.0015 (17)	0.0050 (15)
C18	0.026 (2)	0.022 (2)	0.025 (2)	0.0019 (17)	0.0020 (17)	−0.0034 (16)
C19	0.035 (2)	0.025 (2)	0.022 (2)	0.0052 (18)	0.0060 (18)	−0.0038 (17)
C20	0.036 (2)	0.023 (2)	0.026 (2)	0.0018 (18)	0.0038 (19)	0.0031 (17)
C21	0.038 (2)	0.030 (2)	0.017 (2)	0.0023 (19)	0.0010 (18)	0.0007 (16)
C22	0.045 (3)	0.026 (2)	0.019 (2)	0.0030 (19)	−0.0010 (19)	0.0012 (17)
C23	0.027 (2)	0.021 (2)	0.025 (2)	0.0038 (17)	0.0013 (17)	−0.0003 (16)
C24	0.037 (2)	0.022 (2)	0.024 (2)	0.0015 (18)	−0.0015 (19)	−0.0021 (16)
C25	0.022 (2)	0.026 (2)	0.019 (2)	−0.0033 (16)	0.0018 (16)	0.0029 (16)
C26	0.0127 (19)	0.026 (2)	0.023 (2)	−0.0023 (16)	0.0001 (15)	0.0007 (17)

### Geometric parameters (Å, °)

**Table:**

Cd1—N1	2.246 (3)	C9—H9B	0.9900
Cd1—N3	2.287 (3)	C11—C16	1.374 (5)
Cd1—O2	2.340 (3)	C11—C12	1.391 (5)
Cd1—O1	2.348 (3)	C12—C13	1.360 (5)
Cd1—O3	2.377 (3)	C12—H12A	0.9500
Cd1—O4	2.382 (3)	C13—C14	1.429 (5)
Cd1—C17	2.681 (4)	C13—H13A	0.9500
Cd1—C26	2.729 (4)	C14—C15	1.372 (5)
N1—C7	1.326 (5)	C14—H14A	0.9500
N1—C1	1.393 (5)	C15—C16	1.405 (5)
N2—C7	1.347 (5)	C15—H15A	0.9500
N2—C6	1.380 (5)	C17—C18	1.522 (5)
N2—H2A	0.8800	C18—C19	1.521 (5)
N3—C10	1.325 (5)	C18—H18A	0.9900
N3—C11	1.418 (5)	C18—H18B	0.9900
N4—C10	1.344 (5)	C19—C20	1.518 (5)
N4—C16	1.375 (5)	C19—H19A	0.9900
N4—H4A	0.8800	C19—H19B	0.9900
O1—C17	1.244 (5)	C20—C21	1.525 (6)
O2—C17	1.270 (4)	C20—H20A	0.9900
O3—C26	1.269 (4)	C20—H20B	0.9900
O4—C26	1.252 (4)	C21—C22	1.528 (5)
C1—C6	1.398 (5)	C21—H21A	0.9900
C1—C2	1.401 (5)	C21—H21B	0.9900
C2—C3	1.377 (6)	C22—C23	1.529 (5)
C2—H2B	0.9500	C22—H22A	0.9900
C3—C4	1.400 (6)	C22—H22B	0.9900
C3—H3A	0.9500	C23—C24	1.526 (5)
C4—C5	1.375 (6)	C23—H23A	0.9900
C4—H4B	0.9500	C23—H23B	0.9900
C5—C6	1.400 (6)	C24—C25^i^	1.509 (5)
C5—H5A	0.9500	C24—H24A	0.9900
C7—C8	1.494 (5)	C24—H24B	0.9900
C8—C9	1.519 (5)	C25—C26	1.506 (5)
C8—H8A	0.9900	C25—C24^ii^	1.509 (5)
C8—H8B	0.9900	C25—H25A	0.9900
C9—C10	1.503 (5)	C25—H25B	0.9900
C9—H9A	0.9900		
			
N1—Cd1—N3	98.51 (11)	N4—C10—C9	120.3 (3)
N1—Cd1—O2	145.72 (10)	C16—C11—C12	120.5 (3)
N3—Cd1—O2	102.28 (10)	C16—C11—N3	108.8 (3)
N1—Cd1—O1	92.89 (10)	C12—C11—N3	130.7 (3)
N3—Cd1—O1	104.02 (10)	C13—C12—C11	118.3 (4)
O2—Cd1—O1	55.80 (9)	C13—C12—H12A	120.9
N1—Cd1—O3	102.14 (10)	C11—C12—H12A	120.9
N3—Cd1—O3	86.56 (9)	C12—C13—C14	121.0 (4)
O2—Cd1—O3	106.02 (9)	C12—C13—H13A	119.5
O1—Cd1—O3	160.22 (9)	C14—C13—H13A	119.5
N1—Cd1—O4	94.74 (10)	C15—C14—C13	121.2 (4)
N3—Cd1—O4	141.03 (10)	C15—C14—H14A	119.4
O2—Cd1—O4	85.97 (9)	C13—C14—H14A	119.4
O1—Cd1—O4	111.74 (9)	C14—C15—C16	116.2 (3)
O3—Cd1—O4	54.78 (8)	C14—C15—H15A	121.9
N1—Cd1—C17	118.86 (11)	C16—C15—H15A	121.9
N3—Cd1—C17	106.81 (11)	C11—C16—N4	106.3 (3)
O2—Cd1—C17	28.27 (10)	C11—C16—C15	122.8 (4)
O1—Cd1—C17	27.65 (10)	N4—C16—C15	130.9 (3)
O3—Cd1—C17	133.42 (10)	O1—C17—O2	121.5 (4)
O4—Cd1—C17	98.27 (10)	O1—C17—C18	119.5 (3)
N1—Cd1—C26	97.00 (11)	O2—C17—C18	119.0 (3)
N3—Cd1—C26	114.25 (11)	O1—C17—Cd1	61.1 (2)
O2—Cd1—C26	98.88 (10)	O2—C17—Cd1	60.75 (19)
O1—Cd1—C26	138.34 (10)	C18—C17—Cd1	171.1 (3)
O3—Cd1—C26	27.70 (10)	C19—C18—C17	111.7 (3)
O4—Cd1—C26	27.29 (10)	C19—C18—H18A	109.3
C17—Cd1—C26	119.61 (11)	C17—C18—H18A	109.3
C7—N1—C1	105.3 (3)	C19—C18—H18B	109.3
C7—N1—Cd1	122.8 (3)	C17—C18—H18B	109.3
C1—N1—Cd1	131.4 (2)	H18A—C18—H18B	107.9
C7—N2—C6	107.6 (3)	C20—C19—C18	111.8 (3)
C7—N2—H2A	126.2	C20—C19—H19A	109.3
C6—N2—H2A	126.2	C18—C19—H19A	109.3
C10—N3—C11	104.7 (3)	C20—C19—H19B	109.3
C10—N3—Cd1	133.0 (3)	C18—C19—H19B	109.3
C11—N3—Cd1	119.3 (2)	H19A—C19—H19B	107.9
C10—N4—C16	107.8 (3)	C19—C20—C21	114.8 (3)
C10—N4—H4A	126.1	C19—C20—H20A	108.6
C16—N4—H4A	126.1	C21—C20—H20A	108.6
C17—O1—Cd1	91.2 (2)	C19—C20—H20B	108.6
C17—O2—Cd1	91.0 (2)	C21—C20—H20B	108.6
C26—O3—Cd1	91.8 (2)	H20A—C20—H20B	107.5
C26—O4—Cd1	92.0 (2)	C20—C21—C22	111.6 (3)
N1—C1—C6	109.1 (3)	C20—C21—H21A	109.3
N1—C1—C2	131.1 (4)	C22—C21—H21A	109.3
C6—C1—C2	119.8 (4)	C20—C21—H21B	109.3
C3—C2—C1	117.8 (4)	C22—C21—H21B	109.3
C3—C2—H2B	121.1	H21A—C21—H21B	108.0
C1—C2—H2B	121.1	C21—C22—C23	115.1 (3)
C2—C3—C4	121.3 (4)	C21—C22—H22A	108.5
C2—C3—H3A	119.4	C23—C22—H22A	108.5
C4—C3—H3A	119.4	C21—C22—H22B	108.5
C5—C4—C3	122.4 (4)	C23—C22—H22B	108.5
C5—C4—H4B	118.8	H22A—C22—H22B	107.5
C3—C4—H4B	118.8	C24—C23—C22	112.2 (3)
C4—C5—C6	116.0 (4)	C24—C23—H23A	109.2
C4—C5—H5A	122.0	C22—C23—H23A	109.2
C6—C5—H5A	122.0	C24—C23—H23B	109.2
N2—C6—C1	105.5 (3)	C22—C23—H23B	109.2
N2—C6—C5	131.8 (4)	H23A—C23—H23B	107.9
C1—C6—C5	122.7 (4)	C25^i^—C24—C23	113.3 (3)
N1—C7—N2	112.5 (3)	C25^i^—C24—H24A	108.9
N1—C7—C8	125.9 (4)	C23—C24—H24A	108.9
N2—C7—C8	121.6 (3)	C25^i^—C24—H24B	108.9
C7—C8—C9	115.2 (3)	C23—C24—H24B	108.9
C7—C8—H8A	108.5	H24A—C24—H24B	107.7
C9—C8—H8A	108.5	C26—C25—C24^ii^	117.6 (3)
C7—C8—H8B	108.5	C26—C25—H25A	107.9
C9—C8—H8B	108.5	C24^ii^—C25—H25A	107.9
H8A—C8—H8B	107.5	C26—C25—H25B	107.9
C10—C9—C8	115.5 (3)	C24^ii^—C25—H25B	107.9
C10—C9—H9A	108.4	H25A—C25—H25B	107.2
C8—C9—H9A	108.4	O4—C26—O3	120.5 (3)
C10—C9—H9B	108.4	O4—C26—C25	119.4 (3)
C8—C9—H9B	108.4	O3—C26—C25	120.0 (3)
H9A—C9—H9B	107.5	O4—C26—Cd1	60.71 (19)
N3—C10—N4	112.4 (3)	O3—C26—Cd1	60.53 (19)
N3—C10—C9	127.3 (3)	C25—C26—Cd1	169.2 (3)

Symmetry codes: (i) *x*, −*y*+3/2, *z*−1/2; (ii) *x*, −*y*+3/2, *z*+1/2.

### Hydrogen-bond geometry (Å, °)

**Table:**

*D*—H···*A*	*D*—H	H···*A*	*D*···*A*	*D*—H···*A*
N2—H2A···O2^iii^	0.88	1.85	2.722 (4)	173
N4—H4A···O3^iv^	0.88	1.87	2.686 (4)	154

Symmetry codes: (iii) *x*−1, *y*, *z*; (iv) −*x*, −*y*+1, −*z*+1.
